# Fabrication of a silver particle-integrated silicone polymer-covered metal stent against sludge and biofilm formation and stent-induced tissue inflammation

**DOI:** 10.1038/srep35446

**Published:** 2016-10-14

**Authors:** Tae Hoon Lee, Bong Seok Jang, Min Kyo Jung, Chan Gi Pack, Jun-Ho Choi, Do Hyun Park

**Affiliations:** 1 Department of Internal Medicine, Soonchunhyang University College of Medicine, Cheonan Hospital, Cheonan, South Korea; 2 R&D Institute of Intervention, M. I. Tech Co., Ltd., 174 Habuk 2gil, Jinwi-myeon, Pyeongtaek, Gyeonggi-do, 451-864, South Korea; 3 Department of Convergence Medicine, University of Ulsan College of Medicine & Asan Institute for Life Sciences, Asan Medical Center, Seoul, South Korea; 4 School of Life Sciences and Biotechnology, Korea University, Seoul, Korea; 5 Department of Internal Medicine, Dankook University Hospital, Dankook University College of Medicine, Cheonan, South Korea; 6 Department of Internal Medicine, University of Ulsan College of Medicine, Asan Medical Center, 88 Olympic-ro 43 gil, Songpa-gu, Seoul 05505, South Korea

## Abstract

To reduce tissue or tumor ingrowth, covered self-expandable metal stents (SEMSs) have been developed. The effectiveness of covered SEMSs may be attenuated by sludge or stone formation or by stent clogging due to the formation of biofilm on the covering membrane. In this study, we tested the hypothesis that a silicone membrane containing silver particles (Ag-P) would prevent sludge and biofilm formation on the covered SEMS. *In vitro*, the Ag-P-integrated silicone polymer-covered membrane exhibited sustained antibacterial activity, and there was no definite release of silver ions from the Ag-P-integrated silicone polymer membrane at any time point. Using a porcine stent model, *in vivo* analysis demonstrated that the Ag-P-integrated silicone polymer-covered SEMS reduced the thickness of the biofilm and the quantity of sludge formed, compared with a conventional silicone-covered SEMS. *In vivo*, the release of silver ions from an Ag-P-integrated silicone polymer-covered SEMS was not detected in porcine serum. The Ag-P-integrated silicone polymer-covered SEMS also resulted in significantly less stent-related bile duct and subepithelium tissue inflammation than a conventional silicone polymer-covered SEMS. Therefore, the Ag-P-integrated silicone polymer-covered SEMS reduced sludge and biofilm formation and stent-induced pathological changes in tissue. This novel SEMS may prolong the stent patency in clinical application.

Pancreatic cancer is the fourth leading cause of cancer death, and an estimated 53,070 adults in the United States will be diagnosed with pancreatic cancer in 2016^1^. Among them, 80% are inoperable, owing to disease progression or comorbidities. Since 70% to 85% of patients have tumors involving the head of the pancreas, obstructive jaundice is the common initial presentation. The standard care for the palliation of obstructive jaundice is endoscopic biliary drainage using plastic or metal stents^2^. However, plastic endoprostheses require changing at 3 to 6 months because of occlusion and a return of jaundice, fever, and discomfort. To prolong stent patency and for reasons of cost-effectiveness in unresectable malignant biliary obstruction, self-expandable metal stents (SEMSs), which have a large bore (up to 10 mm diameter), are preferred over plastic stents (up to 3.3 mm diameter)^3^,^4^,^5^. Covered SEMSs have been developed to prevent tumor ingrowth or tissue hyperplasia through the wire mesh, which is a frequent adverse event in uncovered SEMSs. Three commercially available biliary metal stent covering materials are e-PTFE (expanded polytetrafluoroethylene), silicone, and polyurethane^6^. Of these, silicone has less biofilm formation than e-PTFE and is more durable than polyurethane^6^. Therefore, silicone-covered SEMSs are most frequently used. Silicone-covered SEMSs with anti-migration properties improved patency duration without increasing complications, compared with uncovered SEMSs for distal biliary obstruction due to pancreas carcinomas in a randomized multicenter trial^7^. However, stent occlusion in silicone-covered SEMSs can be also caused by biliary sludge, stone formation, or stent clogging owing to bacterial adherence and subsequent biofilm formation on the inner surface of the covered membrane in a stent^7^,^8^,^9^,^10^,^11^.

A biofilm is a group of microorganisms adherent to one another on a surface. Biofilm formation occurs when free-floating microorganisms attach to a surface. They secrete extracellular polymeric substances that provide a structural matrix and facilitate adhesion^12^. Stent blockage affects all types of covered metal stents in current use, and management of this problem requires exchange of the blocked stent on a regular basis—or reinsertion—resulting in increased cost and decreased quality of life^3^. Therefore, the development of a new cover material that minimizes sludge and biofilm formation may be required for longer stent patency^7^.

To prevent biofilm formation on biliary stents, several *in vitro* and *in vivo* animal studies have reported the bactericidal efficacy of silver-coated plastic stents due to the release of silver ions, which suggested prolonged stent patency, but the clinical efficacy has not been evaluated^13^,^14^,^15^,^16^,^17^. Recently, the concept of using silver nanoparticles (AgNPs) as an antimicrobial agent in a plastic stent has been studied^18^,^19^. In malignant biliary obstruction, covered SEMS is the standard treatment, and the role of the plastic stent has been limited owing to the prolonged stent patency of a covered SEMS compared to that of a plastic stent^3^. Theoretically, SEMSs covered with silver may have more prolonged stent patency in terms of the prevention of sludge and biofilm formation. However, a preliminary experiment indicated that it is very difficult to coat a AgNP complex on both the external layer and the inner layer of metal stents^17^.

Colloidal AgNPs tend to form aggregates in the aqueous phase, which gradually diminish their antibacterial efficacy in long-term use. Hence, immobilization of nanoparticles on a surface may be crucial for the durable efficacy of antibacterial function^20^,^21^,^22^. In general, the mode of antibacterial mechanism of AgNPs is attributed to the release of silver ions, which may rupture the cell wall, cause protein denaturation, block cell respiration, and finally cause microbial death^19^. Recently, surface immobilized AgNPs with a contact killing mechanism showed greater efficacy for antibacterial function than colloidal AgNPs, as well as a higher concentration of silver ions in solution^18^,^19^,^22^. Because colloidal AgNPs were found to be sequestered inside the cells and were not accessible to the other cells, bactericidal kinetics may be slower than for the immobilized AgNP with direct contact killing of bacteria^19^. Although the immobilization process of AgNPs could minimize toxicity to mammalian cells by avoiding the excess release of AgNP into solution, the potential cytotoxicity of mobile silver nanoparticles (AgNPs, size 1 to 100 nm) remains a major concern in clinical application^23^,^24^,^25^.

In the biliary tract, the adult human produces about 400 to 800 ml of bile daily. In this aqueous state, the efficacy of a mobile particle or drug-delivery system (e.g., eluting) for antibacterial function may decrease with time. Therefore, immobilization of the particle with antibacterial function in a biliary stent may be essential for the prevention of sludge and biofilm formation.

We hypothesized that a silver particle (Ag-P; fine particle, size 250 nm)-integrated silicone polymer-covered membrane for the immobilization of Ag-P in a covered metal stent may prevent sludge and biofilm formation. Furthermore, the inclusion of Ag-P in the silicone membrane for the entrapment of particles in silicone polymer would likely reduce concerns regarding the release and systemic absorption of Ag-P.

This proof-of-concept *in vitro* and *in vivo* study investigated the efficacy of an Ag-P-integrated, silicone polymer-covered SEMS in terms of preventing or reducing sludge and biofilm formation in a porcine model and the influence on pathological changes and the release of silver ions and systemic absorption.

## Materials and Methods

This animal study was performed in accordance with the rules of the Institutional Animal Care Committee and the National Center of Efficacy Evaluation for the Development of Health Products Targeting Digestive Disorders (NCEED). The Committee on Animal Research at Inha University and the Animal Protection Committee of the Korean Government approved this study. The primary goals of this study were assessment of sludge and biofilm formation and pathological tissue reactivity of an Ag-P-integrated, silicone polymer-covered SEMS compared with a conventional silicone-covered SEMS.

### Materials

#### Fabrication of an Ag-P-integrated silicone polymer-covered self-expandable metal stent

**Figure 1:**
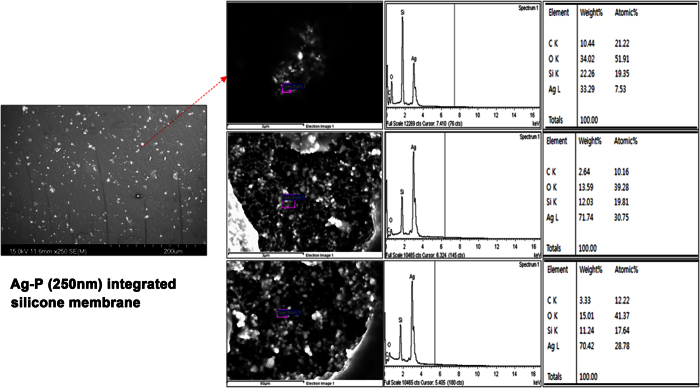
FE-SEM (field emission scanning electron microscope) image & EDX (energy dispersive X-ray spectra) analysis of Ag-P integrated silicone polymer membrane. The SEM image showed a uniformly distributed degree of Ag-P integrated into the silicone polymer membrane. The atomic percent of Ag was investigated with EDX, and the results were detected for Ag atomic element in the silicone membrane, respectively. (Magnification: x5K, x20K, and x30K).

Ag-P with an average particle size of 250 nm (NP-S250) was purchased from NTbase Co. Ltd. (Seoul, Korea). A silicone polymer including Ag-P was prepared using a silicone dispersion (20% [w/v] silicone dispersion in Xylene, MED-6640; NuSil Technology LLC, Carpinteria, CA, USA), diluted from 20% to 13% by adding Xylene. Mixing by 0.1% (w/w) concentration Ag-P in 13% silicone dispersion was performed. Electrospraying of 0.1% (w/w) Ag-P in 13% silicone dispersion with 16 kV, 15 cm of distance, 0.1 mL/min of flow rate, and 500 rpm of rotating drum for Ag-P integrated silicone polymer membrane was performed in bare nitinol wire mesh (Suppl. Fig. 1). The electrosprayed stent was dried in a 35 °C vacuum oven for 30 min, 75 °C vacuum oven for 60 min, and finally 150 °C vacuum oven for 180 min. According to a cross-sectional image, non-aggregated spherical Ag-P particles were integrated uniformly and distributed homogeneously in and onto the silicone polymer under the nitinol wire mesh, and the dispersive degree of Ag-P integrated into the silicone membrane was characterized by SEM-EDX (HITACHI S-2400) (Fig. 1). The average diameter of the Ag-P was ~250 nm, as determined by SEM. Conventional control stents were ordinary silicone polymer-covered SEMSs not containing Ag-P (Suppl. Fig. 2).

#### Ag-P isolation *in vitro* test with time point

We evaluated Ag-P isolation from silicone polymer containing Ag-P. Each Ag-P integrated silicone polymer-covered SEMS (one SEMS with 3 pieces, per each time point [n = 5]) was soaked in a 0.9% (w/v) saline after cutting into 3 pieces in order to increase the surface area of the sample for Ag-P isolation.

The test was separately performed in each sample for 3 h, 6 h, 1 week, 2 weeks, and 4 weeks by shaking in 0.9% saline at 37 °C and 300 rpm. All 3 pieces of SEMS were removed from the saline, and the remaining solution, at the relevant time point, was completely dried in the convection oven at 60 °C. In order to dissolve the isolated Ag-P, nitric acid solution was added. The result was analyzed by inductively coupled plasma-atomic emission spectroscopy (ICP-AES) (Optima 4300DV; Perkin-Elmer, Norwalk, CT, USA).

#### Antimicrobial activity *in vitro* test with time point

The Ag-P integrated silicone membrane was fabricated in the glass petri dish (90 mm in diameter) with equal weight as 5 g of Ag-P integrated silicone polymer and dried in the convection oven at 180 °C for 3 hrs. The final samples were stored in incubation at 60 °C for 97 days, based on the accelerated aging test method.

We evaluated the antimicrobial activity of the Ag-P-integrated silicone polymer at 1 day, and 1, 2, 4, 8, 12, and 24 weeks; a silicone polymer without Ag-P was used as the negative control. *Escherichia coli* and *Klebsiella pneumoniae* were seeded on each plate, either at the initial time or at weekly intervals. The antimicrobial activity test was performed using a JIS Z 2801 (Bioteca Co. Ltd., Palo Alto, CA, USA) as the plate counting method. Antimicrobial activity was calculated as follows: ([Mb-Mc]/Mb) × 100 (Mb: proliferated colony forming unit [CFU] after incubating condition; Mc: control or test CFU).

#### *In vivo* experiments using a porcine model

*In vivo* experiments were performed using 15 (test = 7, control = 7, and negative control = 1) domesticated female mini-pigs with a mean weight of 40 kg (range: 38–42 kg). Before endoscopic procedures, the animals were fasted for 48 h. On the day of the procedure, animals were sedated by an intramuscular injection of tiletamine/zolazepam (Zoletil; 5 mg/kg) and xylazine (Narcoxyl; 2 mg/kg). Anesthesia was administered by intravenous injection (0.1 mg/kg), followed by continuous infusion (4–6 mg/h) of vecuronium. After tracheal intubation, the animals were ventilated with a 1:1 mixture of 1.0–2.0% inspired isoflurane (Ifran®; Hana Pharm Co. Ltd., Seoul, Korea), and oxygen (5–10 mL/kg per min). The animals were placed recumbently on their left sides on a fluoroscopy table. Vital signs were monitored continuously during the procedure. Prophylactic antibiotics were administered 1 day before and after the SEMS insertion. The animals were maintained on their usual diet for 8 weeks following successful stent placement. All animals were euthanized by potassium chloride overdose 8 weeks post-stent insertion.

#### Stent placement and follow-up protocol

Following confirmation of biliary dilation 1 week later (supplementary), randomly numbered pigs underwent endoscopic biliary metal stent (test = 7 and control = 7) deployment through the strictured ampulla of Vater (AV) under fluoroscopy guidance, using a standard duodenoscope (TJF 240; Olympus Optical Co. Ltd., Tokyo, Japan). Following insertion of the SEMSs, the physical status of pigs was monitored daily, and laboratory examinations were performed weekly. The serum Ag level was measured according to a standard protocol. Porcine serum was sampled at 30, 60, and 120 min after stent insertion, and then weekly until pigs were euthanized. Eight weeks after stent insertion, pigs were euthanized and the metal stents harvested for analysis.

### Analysis methods

#### Micro-computed tomography (micro-CT) analysis

**Figure 2:**
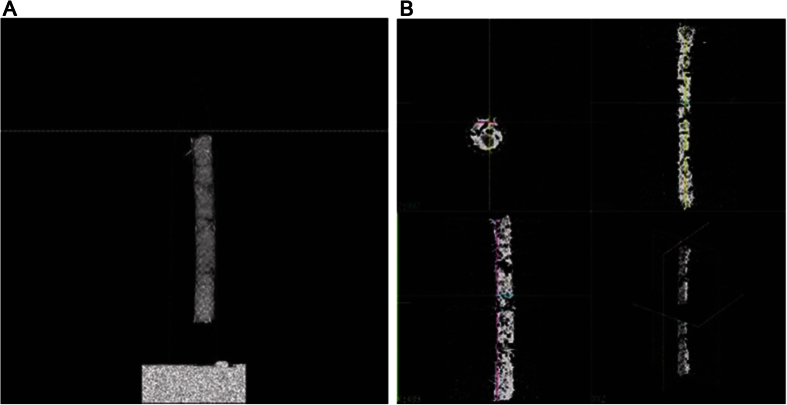
Micro-computed tomography (micro-CT) was performed after treatment of stents with contrast medium and drying in an incubator for 24 h at 36 °C. First, scanning was performed after stent range designation (**A**); then, CT images were analyzed after scanning in the relevant range (**B**).

Imaging at 45 kVp, 88 μA, and 4 W was performed using a micro-CT system (vivaCT 80; Scano Medical AG, Bassersdorf, Switzerland). A standard iodine-based contrast agent (OMNIPAQUE; GE Healthcare, Cork, Ireland) was used in a volume of 100 mL, which contained ~30 g of iodine (300 mg/mL). Micro-CT was performed after treatment of stents with contrast medium and drying in an incubator for 24 h at 36 °C. Then, micro-CT scanning was performed after stent range designation (Fig. 2A). CT images were analyzed after scanning in an appropriate range (Fig. 2B)^26^.

### Image analysis

Stents were dissected longitudinally using an auto surgical blade (Stryker micro dual cut oscillating-sagittal^®^, no. 5400-3-111; Stryker Co., Kalamazoo, MI, USA) and dried in the dark for 24 h at room temperature. Photographs of the stent were obtained using a digital camera (Sony RX100III; Sony Co., Tokyo, Japan). The ratio of dissected internal stent whole area and sludge-stained area was calculated using ImageJ software (ver. 1.48; NIH, Bethesda, MD, USA)^27^,^28^.

### SEM analysis

Biofilm on stents was examined by field-emission scanning electron microscopy (FE-SEM) (S-4700; Hitachi, Tokyo, Japan). This analysis determined the type of bacteria and their spreading pattern on the stent membrane. The stents were washed twice in PBS, and their central portions were cut with scissors along the long axis to a length of 5 × 10 mm. Stents were fixed in 2.5% glutaraldehyde and 4% paraformaldehyde in 0.1 M sodium cacodylate buffer, pH 7.4 for 30 min at 4 °C. Samples were rinsed three times with 0.1 M sodium cacodylate buffer, pH 7.4 for 10 min. After washing, the sample was dehydrated in an ethanol series (50, 70, 80, 90, 95, and 100%) for 20 min each. The stents were critical point-dried using CO_2_, sputter-coated with platinum (Pt), and visualized by FE-SEM^29^.

### Evaluation of biofilm thickness

The stents were washed twice in PBS, and the silicone polymer was cut at 3 randomly selected positions using scissors. Silicone polymer was fixed in 2.5% glutaraldehyde and 4% paraformaldehyde in 0.1 M sodium cacodylate buffer, pH 7.4, for 30 min at 4 °C. Samples were rinsed three times with 0.1 M sodium cacodylate buffer, pH 7.4, for 10 min each. After washing, samples were dehydrated in an ethanol series (50, 70, 80, 90, 95, and 100%) for 20 min each and embedded in Spurr’s resin. After the resin had hardened, ~1 μm thick sections were cut from sample blocks using a diamond knife and ultramicrotome (Ultracut UCT; Leica, Wetzlar, Germany). The sections were flattened by floating on the surface of water at room temperature, collected on clean microscope slides, and dried on a hotplate at 60 °C. The slides were stained with 2% toluidine blue. All images were acquired using a light microscope. Mean biofilm thickness was calculated from 3 sample blocks (20 positions in each sample section) and evaluated using the ImageJ software.

### Serum silver concentration

The serum silver concentration was determined by inductively coupled plasma optical emission spectrometry (ICP-OES) (OPTIMA 8300; Perkin-Elmer) and a microwave (Titan MPS^™^; Perkin-Elmer). The detection limit of this instrument was 0.01 mg/L. The test was performed according to *KS M 0032 (2009): General rules for ICP emission spectrochemical analysis* (K-BIO, Osong Medical Innovation Foundation).

### Histopathologic analysis

After gross inspection of the harvested biliary tracts, tissues were fixed in formalin for subsequent processing and stained with hematoxylin and eosin (H&E) for histological analysis. The specimens were embedded from the stent to minimize potential artifacts from their removal. Then, 50- to 100-μm sections were obtained ~1 mm apart. Inflammation, necrosis, congestion, neovascularization, fibrosis, and mucous hyperplasia of biliary tissue were estimated from grades 0 to 4 as follows: 0 (*not remarkable*); 1 (*minimal*); 2 (*mild*); 3 (*moderate*); and 4 (*severe*). Pathologic review was performed blindly by an experienced third-party pathologist (Korea Conformity Laboratories).

Eight weeks after deployment of SEMSs, porcine biliary tracts, including the metal stents, were harvested. The sludge and biofilm formation was evaluated by micro-CT, ImageJ, and SEM analysis, and tissue pathology was assessed using the above scoring system.

### *In vitro* anti-inflammatory cytokine interleukin (IL)-10 analysis

#### Cell culture

RAW264.7 cells were obtained from the American Type Culture Collection ([ATCC], Manassas, VA, USA). Cells were cultured in Dulbeco’s Modified Eagle’s Medium (DMEM) supplemented with 10% fetal bovine serum (FBS) and antibiotic solution (100 units/mL penicillin and 100 μg/mL streptomycin) at 37 °C in a humidified 5% CO_2_ atmosphere. For further analysis, RAW264.7 cells (1 × 10^6^ cells) were cultured for 72 h in a 100 mm culture dish, coated with silicone only, and Ag-particle integrated silicone polymer membrane (0.01%, 0.1%, and 1), respectively.

#### Ribonucleic acid (RNA) extraction and reverse transcription polymerase chain reaction (RT-PCR)

Total RNA was isolated using a commercially available kit (Nucleospin RNAII, Macherey-Nagel, Germany), following the manufacturer’s instructions. Total 500 ng RNA was reverse-transcribed, and IL-10 and GAPDH transcripts were amplified using an RT-PCR PreMIX Kit (Intron, Inc., Daejon, Korea). The following primer sets were used for RT-PCR: *IL-10* forward, 5′-TAC AGC CGG GAA GAC AAT AAC-3′; *IL-10* reverse, 5′-CAC CTT GGT CTT GGA GCT TAT-3′; *GAPDH* forward, 5′-ACC ACA GTC CAT GCC ATC AC-3′; *GAPDH* reverse, 5′-TCC ACC ACC CTG TTG CTG TA-3′. The PCR reaction was performed in a total volume of 20 μl, and denaturation was run for 35 cycles at 94 °C for 45 sec, annealing at 58 °C for 45 sec, and extension at 72 °C for 1 min, followed by final extension at 72 °C for 5 min.

#### Statistical analysis

The results were presented as mean ± standard deviation (SD). The differences in average of data were compared using the Mann-Whitney U test or independent sample t-test. A value of P < 0.05 was assumed to be statistically significant. All analyses were performed using SPSS version 17.0 (SPSS, Inc., Chicago, IL, USA).

## Results

### *In vitro* Ag-P isolation test in Ag-P integrated silicone polymer-covered metal stent

**Figure 3:**
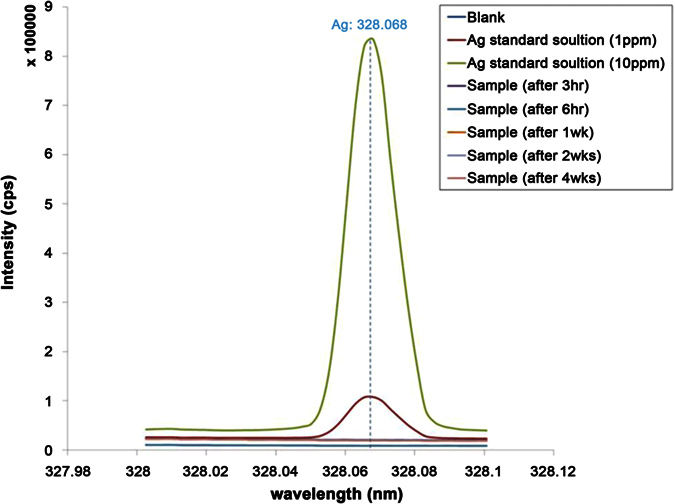
Ag-P isolation over time. The test was performed after 3 and 6 h, and 1, 2, and 4 weeks in 0.9% saline at 37 °C with shaking at 300 rpm. Compared with Ag standard solution (1 and 10 ppm), there was no release of silver ions detected below the detection limit of this instrument in all the samples with Ag-P integrated silicone polymer-covered metal stents.

In the Ag-P isolation test with time points (for 3 h, 6 h, 1 week, 2 weeks, and 4 weeks, by shaking in 0.9% saline at 37 °C and 300 rpm), there was no release of silver ions below the detection limit of this instrument in all the samples with an Ag-P integrated silicone polymer-covered metal stent, compared with Ag standard solution (1 and 10 ppm) (Fig. 3).

### *In vitro* antibacterial activity

**Figure 4:**
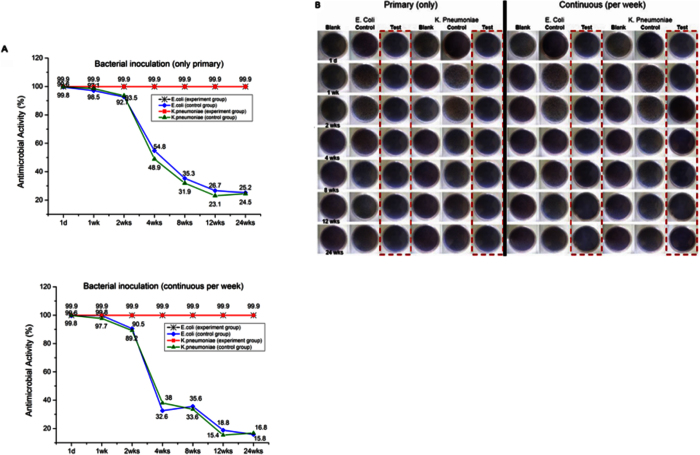
Antimicrobial activity over time. Antimicrobial activity of stents in the test and control groups was evaluated at 1 day, and 1, 2, 4, 8, 12, and 24 weeks, after placement. The test was performed by seeding at the initial time only or at weekly intervals. The result showed a significant difference in antimicrobial activity over time in the silicone polymer membranes with and without Ag-P. The test group showed 99.9% antimicrobial activity against *E. coli* and *K. pneumoniae* over 24 weeks. In contrast, antimicrobial activity was decreased dramatically after 2 weeks in the control group (**A**). Images for inoculation of culture media also showed marked antibacterial activity in the test group (**B**).

The antimicrobial activity of silicone polymer with/without Ag-P differed significantly over time. The test group showed 99.9% antimicrobial activity against *E. coli* and *K. pneumoniae* over 24 weeks. In contrast, antimicrobial activity in the control group decreased dramatically after 2 weeks (Fig. 4A). Qualitative analysis also showed significant inhibition of bacterial growth in the test group (Fig. 4B).

### *In vivo* sludge formation

**Figure 5:**
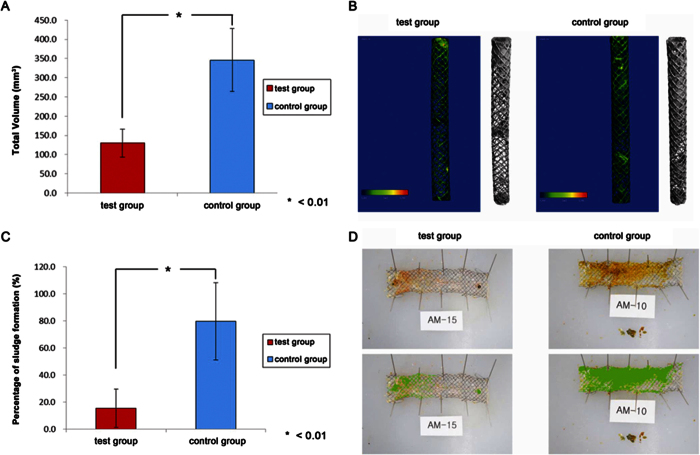
(**A**) Quantitative sludge analysis by micro-CT. The test group exhibited a significantly lower total sludge volume than the control group (P < 0.01). (**B**) Total sludge volume in the test and control groups measured by micro-CT. The value (95.20 mm^3^) of the test group (AM-15, no. 5 in Table 1) was significantly lower than that (301.38 mm^3^) of the control group (AM-10, no. 4 in Table 1) (average P = 0.001). (**C**) Quantitative sludge analysis using ImageJ software. The test group exhibited a significantly lower percentage of sludge formation than the control group (P < 0.01). (**D**) Sludge quantitative analysis. Proportion of sludge formation in the test and control groups is shown. The value (5.3%) of the test group (AM-15) was significantly lower than that (41.0%) of the control group (AM-10) (average P < 0.01).

Quantitative analysis showed significantly lower sludge formation in the test group. In micro-CT analysis, the total measured mean volume (standard deviation, SD) was 129.96 (36.87) mm^3^ in the test group and 345.90 (81.98) mm^3^ in the control group (P < 0.01) (Table 1; Fig. 5A,B). In ImageJ analysis, the mean (SD) proportion of the total area sludge-stained was 15.5% (14.1) in the test group and 79.7% (28.4) in the control group (P < 0.01) (Fig. 5C,D).

### *In vivo* SEM analysis and evaluation of biofilm thickness

**Figure 6:**
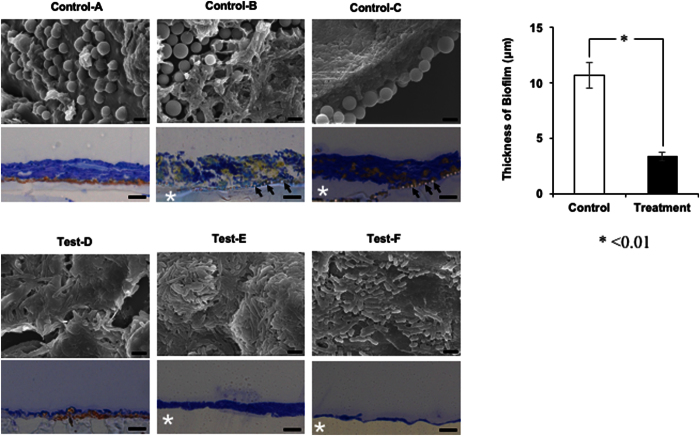
SEM and light microscopy images of biofilm on the inner surface of 6 stents. SEM images of biofilm on 3 stents in the control (**A–C**) and test (**D–F**) groups (original magnification, ×6,000; bar = 2 μm). Biofilm was attached to the silicone membrane (*). Control group stents exhibited heterogeneous biofilm consisting of both spherical (diameter 2.0 ± 0.28 μm) and rod-shaped bacteria, and showed two biofilm patterns. One comprised a layer of spherical bacteria beneath a layer of rod-shaped bacteria (**B,C**). The second comprised spherical bacteria among the rod-shaped bacteria (**A**). In the test group, homogeneous and amorphous biofilm comprising only rod-shaped bacteria was observed. Light micrographs are cross-sectional views of the silicone membrane and biofilm (original magnification × 400, bar = 10 μm). Black arrow indicates spherical bacteria (**B,C**). Mean (SD) biofilm thickness was 3.32 (0.38) μm in the test group and 10.62 (1.15) μm in the control group (P < 0.01).

To evaluate biofilm formation, the luminal surface of stents was visualized by SEM. The luminal surfaces of stents in the control and test groups were covered with biofilm. Rod-shaped and coccoid bacterial cells could be morphologically distinguished. In the control group, heterogeneous biofilm consisting of both coccoid and rod-shape cells was observed. In contrast, homogeneous biofilm comprising only rod-shaped bacteria was observed in the test group. The mean (SD) biofilm thickness was 3.32 (0.38) μm in the test group and 10.62 (1.15) μm in the control group (P < 0.01) (Fig. 6).

### *In vivo* pathologic analysis

**Figure 7:**
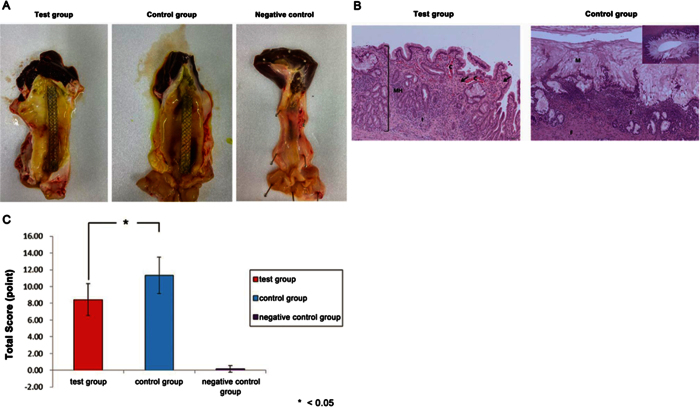
Pathological analysis. (**A**) Gross findings. Compared with the negative control, the test tissue sample showed mild erythematous mucosal change and a normal SEMS configuration. In the control group, more severe erythematous change and bile-clogged membrane stent were noted. (**B**) Microscopic findings. The test group showed minimal mucosal hyperplasia (MH), necrosis (arrow), inflammation (I), and congestion (C) (left; HE stain, ×100). The control group showed remarkable mucosal hyperplasia with mucous secretion (M), fibrosis (F), and inflammation (I) (right; HE stain, ×100 and ×40 in the right upper panel). (**C**) Total pathology score was significantly lower in the test group.

Tissue reactivity was evaluated in the test, control, and normal control groups. Normal control pigs did not show any significant change in tissue. The bile duct in the test group showed minimal erythematous change around the stent and sludge formation on the covered membrane, compared with the control group (Fig. 7A). The test and control groups showed inflammation, necrosis, congestion, neovascularization, fibrosis, and mild to severe mucous hyperplasia in the bile duct epithelium and subepithelium tissue (Fig. 7B). The total mean score (SD) was 8.43 (1.90) in the test group and 11.33 (2.16) in the control group (P = 0.025; Fig. 7C, Table 1).

### *In vitro* IL-10 analysis

Increased and dose-dependent expression of IL-10 in silver particle integrated silicone polymer membrane compared with the control groups (a culture dish without surface coat and a dish coated with silicone membrane alone) was shown in mouse RAW264.7 macrophages (Suppl. Fig. 3).

### *In vivo* safety

Following technically successful metal stent deployment, there was no stent-related mortality during the 8-week study period. No abnormal behavior was detected during daily physical examination. Silver ion was not detected in the serum of all pigs during the follow-up period.

## Discussion

Biliary silicone polymer-covered SEMS occlusion is caused by the deposition of biliary sludge or stone formation owing to bacterial adherence and subsequent biofilm formation^30^. In their clinical observational study, Kida *et al*.^31^ reported that the occlusion rate of silicone polymer-covered SEMS due to biliary sludge or stone formation was 37%.

To prevent biofilm formation on silicone polymer-covered SEMSs, several methods have been proposed, including the use of antibiotics to inhibit bacterial adhesion. An *in vitro* study reported a sustained reduction in *E. coli* adherence with prolonged ciprofloxacin perfusion^32^. Moreover, ampicillin/sulbactam-treated silicone covered SEMSs showed no biofilm formation *in vitro*^30^. Antibiotics may thus prevent bacterial adherence. However, the results of long-term use were disappointing. This may be related to antibiotic resistance and emergence of other gram-positive or anaerobic bacteria species during prolonged use of antibiotics^32^,^33^,^34^. To overcome this problem, antibiotic-eluting metal stents have been developed. However, a study of a cefotaxime-eluting silicone-covered SEMS reported no reduction in biofilm formation in a canine model^35^.

This is the first report of an Ag-P-integrated silicone polymer for the prevention of sludge and biofilm formation and stent-induced tissue inflammation in a covered SEMS. Silver ions have a broad spectrum of antimicrobial activity, although the underlying mechanism is unclear. Cytoplasmic membrane and DNA damage by silver ions are possibilities^13^,^14^. In this study, the Ag-P-integrated silicone membrane inhibited the growth of *E*. *coli* and *K*. *pneumoniae* for >6 months *in vitro* (Fig. 4). Rod-shaped cells were attached to the silicone membrane only in the test group; in the control group, cocci and rods were present on stents. In a previous study, gram negative *E. coli* were more adherent than gram-positive *Enterococcus* in stents. Furthermore, precolonization with *E. coli* facilitated the subsequent attachment of *Enterococcus*^36^. Ag-P integrated silicone polymer-covered SEMSs may inhibit the growth of rods and further adhesion of cocci to rods on the silicone membrane, owing to contact-mode action by the Ag-P in silicone polymer-covered membrane. This may reduce sludge and biofilm formation, as confirmed by micro-CT and image analyses.

A silver-coated plastic stent significantly reduced bacterial adherence by 10- to 100-fold in a dose-dependent manner *in vitro*^15^. An *in vivo* animal study revealed that coating of a polyurethane stent with silver nanoparticles inhibited bacterial adhesion for 7 days^16^. In another study, a silver-coated biliary plastic stent prolonged the unobstructed period, owing to its antibacterial activity^17^. These results suggested that coating of plastic stents with silver nanoparticles resulted in the release of nano-silver, which inhibited bacterial growth and prolonged stent patency.

However, plastic stents have a limited working diameter compared with SEMSs. Also, a concern with the use of silver-coated plastic stents in the human biliary system is systemic absorption of silver nanoparticles released into bile. The safety of silver nanoparticle (<100 nm diameter) accumulation in serum was not proved conclusively in a previous study^37^. Ag-P integrated silicone polymer-covered SEMS by the entrapment of particles in the silicone polymer may have an effect on the immobilization of Ag-P. This may prevent the systemic absorption of Ag-P. In our *in vivo* study, Ag-P particles were not detected in serum, and our *in vitro* results with an aggressive isolation test (3 h, 6 h, 1 week, 2 weeks, and 4 weeks, by shaking in 0.9% saline at 37 °C and 300 rpm in 3 cut pieces of Ag-P integrated silicone polymer-covered SEMS) showed no definite release of silver ions from Ag-P integrated silicone polymer. This condition simulating the crack in the silicone covering membrane in human bile over time may also support the durability and safety of the Ag-P-integrated silicone polymer-covering membrane with time.

Our findings suggest another benefit regarding stent-related inflammation of the bile duct. Stent-related ductal strictures or tissue hyperplasia following removal of silicone-covered SEMSs are an important issue^38^,^39^,^40^. This stent-induced ductal change and epithelial hyperplasia may be related to the ischemic change by excessive outward radial pressure of the SEMS and the damage to the silicone membrane during stent deployment or stent expansion through folding^40^. To evaluate stent-related tissue change, we assessed inflammation, necrosis, congestion, neovascularization, fibrosis, and mucosal hyperplasia of the bile duct. The tissue hyperplasia, neovascularization, and fibrosis scores were significantly lower in the test group than in the control group. The total pathological tissue score was also significantly lower in the test group. Thus, the Ag-P-integrated silicone polymer membrane did not increase reactive tissue changes compared to a conventional silicone polymer membrane. Because silicone covered SEMS in the control group was similar to conventional silicone covered SEMS in terms of material properties, SEMS in the test group may have an improved biocompatibility between the stent and surrounding tissue compared with the biocompatibility of conventional silicone covered SEMS.

In *in vitro* analysis, increased and dose-dependent expression of interleukin (IL)-10 in silver particle integrated silicone polymer membrane compared with control groups was found (Suppl. Fig. 3). This result suggests that the increased activity of IL-10 have a role in the anti-inflammatory effect of silver particle integrated silicone polymer membrane, even though the immobilization of silver in silicone polymer and the lack of release of silver ions.

Further study of the anti-inflammatory of Ag-P integrated silicone-covered SEMSs in various organs—such as the ureter, pancreatic ducts, esophagus, or vascular system—is warranted. Moreover, the integration of other biocompatible metal particles (e.g., copper) in the silicone polymer of a SEMS as the method of the immobilization of metal particles in silicone polymer with the lack of relsease of metal particle may be evaluated for long-term antimicrobial activity.

Ag-P integrated silicone polymer-covered SEMS resulted in reduced sludge and biofilm formation and stent-induced tissue inflammation compared with a conventional silicone-covered SEMS in a porcine model. These characteristics may lead to the prolongation of stent patency in malignant biliary obstruction and reduce the risk of cholangitis, which requires frequent reintervention. Given our favorable preclinical data, further human clinical trials comparing this novel covered and conventional covered SEMS may be encouraged.

## Additional Information

**How to cite this article**: Lee, T. H. *et al*. Fabrication of a silver particle-integrated silicone polymer-covered metal stent against sludge and biofilm formation and stent-induced tissue inflammation. *Sci. Rep.* **6**, 35446; doi: 10.1038/srep35446 (2016).

## Supplementary Material

Supplementary Information

## Figures and Tables

**Table 1:** Histopathological scores of the porcine biliary tract mucosa.

Score, mean ± SD	Test	Control	*P* value
Inflammation	1.14 ± 0.38	1.17 ± 0.41	0.915
Necrosis	1.43 ± 1.13	1.33 ± 1.03	0.878
Congestion	0.29 ± 0.49	0.67 ± 0.52	0.199
Neovascularization	1.57 ± 0.53	2.50 ± 0.55	0.010
Fibrosis	1.71 ± 0.49	2.50 ± 0.55	0.019
Mucosal hyperplasia	2.29 ± 0.49	3.17 ± 0.41	0.005
Total scores	8.43 ± 1.90	11.33 ± 2.16	0.025

SD, standard deviation.

## References

[b1] Siegel R. L., Miller K. D. & Jemal A. Cancer statistics, 2016. CA Cancer J. Clin. 66, 7–30, 10.3322/caac.21332 (2016). 26742998

[b2] Brescia F. J. Palliative care in pancreatic cancer. Cancer Control 11, 39–45 (2004). 14749622 10.1177/107327480401100206

[b3] Walter D. . Cost efficacy of metal stents for palliation of extrahepatic bile duct obstruction in a randomized controlled trial. Gastroenterology 149, 130–138, 10.1053/j.gastro.2015.03.012 (2015). 25790742

[b4] Lee J. H. Self-expandable metal stents for malignant distal biliary strictures. *Gastrointest.* Endosc. Clin. N. Am. 21, 463–480, viii-ix, 10.1016/j.giec.2011.04.009 (2011). 21684465

[b5] Abraham N. S., Barkun J. S. & Barkun A. N. Palliation of malignant biliary obstruction: a prospective trial examining impact on quality of life. Gastrointest. Endosc. 56, 835–841, 10.1067/mge.2002.129868 (2002). 12447294

[b6] Bang B. W. . The biodurability of covering materials for metallic stents in a bile flow phantom. Dig. Dis. Sci. 57, 1056–1063, 10.1007/s10620-011-1958-6 (2012). 22101941

[b7] Kitano M. . Covered self-expandable metal stents with an anti-migration system improve patency duration without increased complications compared with uncovered stents for distal biliary obstruction caused by pancreatic carcinoma: a randomized multicenter trial. Am. J. Gastroenterol. 108, 1713–1722, 10.1038/ajg.2013.305 (2013). 24042190

[b8] Leung J. W., Ling T. K., Kung J. L. & Vallance-Owen J. The role of bacteria in the blockage of biliary stents. Gastrointest. Endosc. 34, 19–22 (1988). 3280393 10.1016/s0016-5107(88)71223-7

[b9] Speer A. G. . Biliary stent blockage with bacterial biofilm. A light and electron microscopy study. Ann. Intern. Med. 108, 546–553 (1988). 2450501 10.7326/0003-4819-108-4-546

[b10] Sung J. J. Bacterial biofilm and clogging of biliary stents. J. Ind. Microbiol. 15, 152–155 (1995). 8519471 10.1007/BF01569819

[b11] Costerton J. W., Montanaro L. & Arciola C. R. Biofilm in implant infections: its production and regulation. Int. J. Artif. Organs 28, 1062–1068 (2005). 16353112 10.1177/039139880502801103

[b12] Wellman N., Fortun S. M. & McLeod B. R. Bacterial biofilms and the bioelectric effect. Antimicrob. Agents Chemother. 40, 2012–2014 (1996). 8878572 10.1128/aac.40.9.2012 PMC163464

[b13] Radzig M. A. . Antibacterial effects of silver nanoparticles on gram-negative bacteria: influence on the growth and biofilms formation, mechanisms of action. Colloids Surf. B. Biointerfaces 102, 300–306, 10.1016/j.colsurfb.2012.07.039 (2013). 23006569

[b14] Feng Q. L. . A mechanistic study of the antibacterial effect of silver ions on Escherichia coli and Staphylococcus aureus. J. Biomed. Mater. Res. 52, 662–668 (2000). 11033548 10.1002/1097-4636(20001215)52:4<662::aid-jbm10>3.0.co;2-3

[b15] Leung J. W., Lau G. T., Sung J. J. & Costerton J. W. Decreased bacterial adherence to silver-coated stent material: an *in vitro* study. Gastrointest. Endosc. 38, 338–340 (1992). 1607086 10.1016/s0016-5107(92)70428-3

[b16] Wen W. . Silver-nanoparticle-coated biliary stent inhibits bacterial adhesion in bacterial cholangitis in swine. Hepatobiliary Pancreat. Dis. Int. 15, 87–92 (2016). 26818548 10.1016/s1499-3872(15)60410-6

[b17] Yang F. . A novel biliary stent coated with silver nanoparticles prolongs the unobstructed period and survival via anti-bacterial activity. Sci. Rep. 6, 21714, 10.1038/srep21714 (2016). 26883081 PMC4756318

[b18] He M., Lu L., Zhang J. & Li D. Immobilized silver nanoparticles on chitosan with special surface state-enhanced antimicrobial efficacy and reduced cytotoxicity. J. Nanosci. Nanotechnol. 15, 6435–6443 (2015). 26716197 10.1166/jnn.2015.10782

[b19] Agnihotri S. & Mukherji S. Immobilized silver nanoparticles enhance contact killing and show highest efficacy: elucidation of the mechanism of bactericidal action of silver. Nanoscale 5, 7328–7340, 10.1039/c3nr00024a (2013). 23821237

[b20] Cao H., Liu X., Meng F. & Chu P. K. Biological actions of silver nanoparticles embedded in titanium controlled by micro-galvanic effects. Biomaterials 32, 693–705, 10.1016/j.biomaterials.2010.09.066 (2011). 20970183

[b21] Qin H. . *In vitro* and *in vivo* anti-biofilm effects of silver nanoparticles immobilized on titanium. Biomaterials 35, 9114–9125, 10.1016/j.biomaterials.2014.07.040 (2014). 25112937

[b22] Taheri S. . Synthesis and surface immobilization of antibacterial hybrid silver-poly(l-lactide) nanoparticles. Nanotechnology 25, 305102, 10.1088/0957-4484/25/30/305102 (2014). 25007946

[b23] Loher S., Schneider O. D., Maienfisch T., Bokorny S. & Stark W. J. Micro-organism-triggered release of silver nanoparticles from biodegradable oxide carriers allows preparation of self-sterilizing polymer surfaces. Small 4, 824–832, 10.1002/smll.200800047 (2008). 18416429

[b24] AshaRani P. V., Low Kah Mun G., Hande M. P. & Valiyaveettil S. Cytotoxicity and genotoxicity of silver nanoparticles in human cells. ACS Nano 3, 279–290, 10.1021/nn800596w (2009). 19236062

[b25] Pratsinis A., Hervella P., Leroux J. C., Pratsinis S. E. & Sotiriou G. A. Toxicity of silver nanoparticles in macrophages. Small 9, 2576–2584, 10.1002/smll.201202120 (2013). 23418027

[b26] Morlacchi S. . Hemodynamics and in-stent restenosis: micro-CT images, histology, and computer simulations. Ann. Biomed. Eng. 39, 2615–2626, 10.1007/s10439-011-0355-9 (2011). 21785884

[b27] Jensen E. C. Quantitative analysis of histological staining and fluorescence using ImageJ. Anat. Rec. (Hoboken) 296, 378–381, 10.1002/ar.22641 (2013). 23382140

[b28] Collins T. J. ImageJ for microscopy. Biotechniques 43, 25–30 (2007). 10.2144/000112517 17936939

[b29] van Berkel A. M., van Marle J., van Veen H., Groen A. K. & Huibregtse K. A scanning electron microscopic study of biliary stent materials. Gastrointest. Endosc. 51, 19–22 (2000). 10625789 10.1016/s0016-5107(00)70380-4

[b30] Tsang T. K., Pollack J. & Chodash H. B. Silicone-covered metal stents: an *in vitro* evaluation for biofilm formation and patency. Dig. Dis. Sci. 44, 1780–1785 (1999). 10505714 10.1023/a:1018826202753

[b31] Kida M. . Endoscopic management of malignant biliary obstruction by means of covered metallic stents: primary stent placement vs. re-intervention. Endoscopy 43, 1039–1044, 10.1055/s-0030-1256769 (2011). 21971926

[b32] Leung J. W. . *In vitro* evaluation of antibiotic prophylaxis in the prevention of biliary stent blockage. Gastrointest. Endosc. 51, 296–303 (2000). 10699774 10.1016/s0016-5107(00)70358-0

[b33] Libby E. D. & Leung J. W. Prevention of biliary stent clogging: a clinical review. Am. J. Gastroenterol. 91, 1301–1308 (1996). 8677983

[b34] Luman W., Ghosh S. & Palmer K. R. A combination of ciprofloxacin and Rowachol does not prevent biliary stent occlusion. Gastrointest. Endosc. 49, 316–321 (1999). 10049414 10.1016/s0016-5107(99)70007-6

[b35] Gwon D. I., Lee S. S. & Kim E. Y. Cefotaxime-eluting covered self-expandable stents in a canine biliary model: scanning electron microscopic study of biofilm formation. Acta Radiol. 53, 1127–1132, 10.1258/ar.2012.120220 (2012). 23034797

[b36] Leung J. W. . Is there a synergistic effect between mixed bacterial infection in biofilm formation on biliary stents? Gastrointest. Endosc. 48, 250–257 (1998). 9744599 10.1016/s0016-5107(98)70186-5

[b37] Schluesener J. K. & Schluesener H. J. Nanosilver: application and novel aspects of toxicology. Arch. Toxicol. 87, 569–576, 10.1007/s00204-012-1007-z (2013). 23344422

[b38] Oh D. . Feasibility and safety of a fully covered self-expandable metal stent with antimigration properties for EUS-guided pancreatic duct drainage: early and midterm outcomes (with video). Gastrointest. Endosc. 83, 366–373 e362, 10.1016/j.gie.2015.07.015 (2016). 26324387

[b39] Moon S. H. . Modified fully covered self-expandable metal stents with antimigration features for benign pancreatic-duct strictures in advanced chronic pancreatitis, with a focus on the safety profile and reducing migration. Gastrointest. Endosc. 72, 86–91, 10.1016/j.gie.2010.01.063 (2010). 20493483

[b40] Lee S. S. . Histological changes in the bile duct after long-term placement of a fully covered self-expandable metal stent within a common bile duct: a canine study. Clin. Endosc. 47, 84–93, 10.5946/ce.2014.47.1.84 (2014). 24570888 PMC3928498

