# Supplementary material for: Fabrication of a silver particle-integrated silicone polymer-covered metal stent against sludge and biofilm formation and stent-induced tissue inflammation
Source: Sci Rep. 2016 Oct 14;6:35446. doi: 10.1038/srep35446 (PMC5064322; doi:10.1038/srep35446)
Supplement: Supplementary Information [file srep35446-s1.pdf]

Fabrication of a silver particle-integrated silicone polymer-covered metal stent against sludge and biofilm formation and stent-induced tissue inflammation

Tae Hoon Lee, MD, PhD, Bong Seok Jang, MS, Min Kyo Jung, MS, Chan Gi Pack, PhD, Jun-Ho Choi, MD, PhD, Do Hyun Park, MD, PhD

Supplementary method

Preparation of the experimental biliary dilatation porcine model and stent insertion

Prior to stent insertion, we generated a porcine stricture and biliary dilatation model by endoscopic band ligation of the AV, followed by argon plasma coagulation (APC System, power setting 40 W, gas flow 2 L/min; ERBE Elektromedizin GmbH, Tubingen, Germany) in the orifice or AV based on our previous report [1]. One week later, biliary dilation was confirmed by endoscopic retrograde cholangiography (ERC), followed by deployment of the metal stent across the AV. One week later, the pigs underwent endoscopic biliary metal stent deployment through the strictured AV. Following insertion of metal stents, the physical status and laboratory parameters of the pigs were monitored daily. Eight weeks later,m animals were euthanized and the metal stents harvested for analysis.

Reference

[1] T.H. Lee, J.H. Choi, S.S. Lee, H.D. Cho, D.W. Seo, S.H. Park, S.K. Lee, M.H. Kim, D.H. Park, A pilot proof-of-concept study of a modified device for one-step endoscopic ultrasound-guided biliary drainage in a new experimental biliary dilatation animal model, World J. Gastroenterol. 20 (2014) 5859-5866.

Supplementary Fig.1

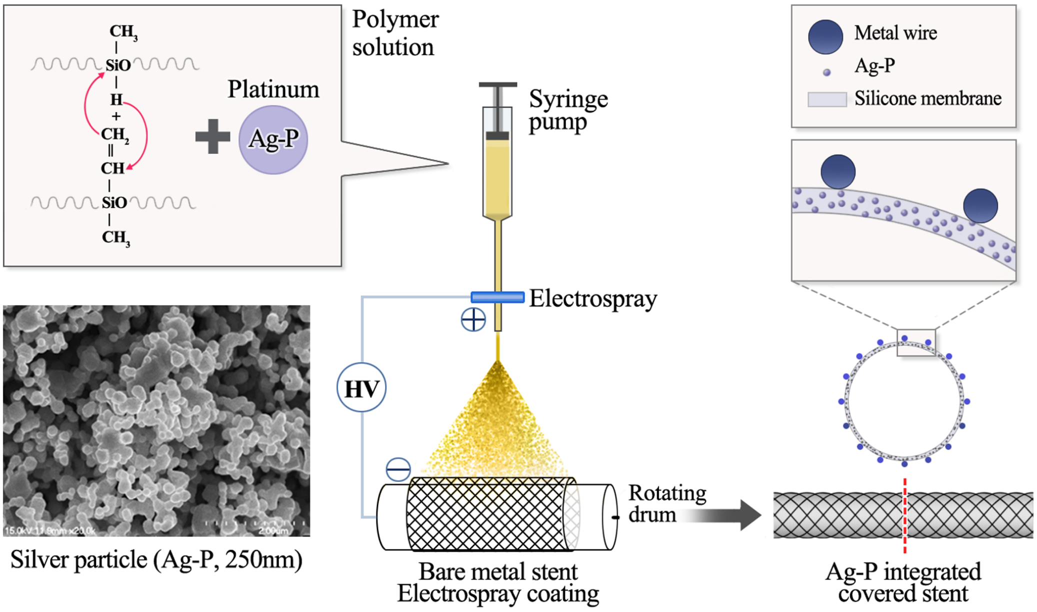

Supplementary Fig 2

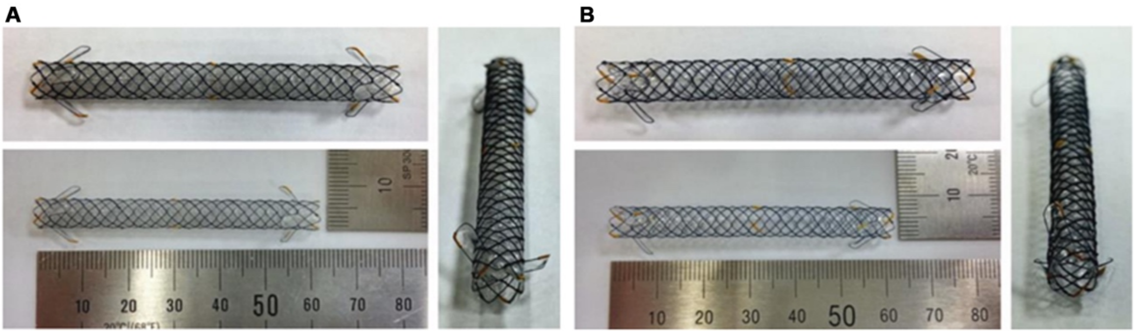

Supplementary Fig.3

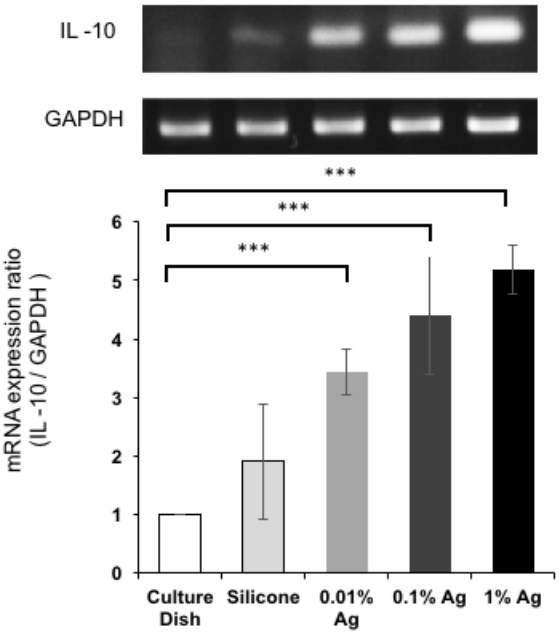

Supplementary Fig.1. A silicone polymer including Ag-P for the prevention of sludge and biofilm formation was prepared using a silicone dispersion (silicone dispersion in Xylene, Ag-P [250nm], and platinum as a catalyst). Electrospraying of 0.1% (w/w) Ag-P in 13% silicone dispersion with 16 kV, 15cm of distance, 0.1 mL/min of flow rate, and 500 rpm of rotating drum for Ag-P integrated silicone polymer membrane was performed in bare nitinol wire mesh.

Supplementary Fig.2. Stent morphology (A, test group; B, control group). The morphology of fully silicone covered self-expandable metal stents (SEMS) with three flaps in both ends (6 mm diameter and 60 mm length) did not differ between the test and control groups.

Supplementary Fig.3**.** Ag integrated silicone membrane induce anti-inflammatory cytokine

interleukin (IL)-10 in mouse Raw264.7 macrophages.

Mouse Raw264.7 macrophages (1 x 10^6 cells/dish) were seeded in a 100 mm culture dish without surface coat, in a dish coated with silicone membrane alone, and in dishes coated with 0.01%, 0.1%, and 1% Ag-particle integrated silicone membrane, respectively.

Each bar represents the mean ± standard deviation of samples run in triplication. The data are representative of three independent experiments; significantly different from culture dish ($P < 0.005$)
